# The Influence of Saliva pH on the Fracture Resistance of Two Types of Implant-Supported Bis-Acrylic Resin Provisional Crowns—An In Vitro Study

**DOI:** 10.3390/jfb14020062

**Published:** 2023-01-21

**Authors:** Sofia Sousa-Santos, António Sérgio Silva, Primavera Sousa-Santos, Teresa Vale, José Manuel Mendes

**Affiliations:** 1 Department of Dental Sciences, University Institute of Health Sciences (IUCS), CESPU, 4585-116 Gandra, Portugal; 2 UNIPRO—Oral Pathology and Rehabilitation Research Unit, University Institute of Health Sciences (IUCS), CESPU, 4585-116 Gandra, Portugal

**Keywords:** fracture resistance, bite force, CAD/CAM, implant provisional restorations, bis-acrylic resins, saliva pH

## Abstract

Temporary restorations play a fundamental role in oral rehabilitation. A properly adapted implant-supported provisional restoration implies better esthetics, contouring and architectural modeling of the upper peri-implant tissue. This study aimed to evaluate the influence of oral pH on the fracture resistance of implant-supported provisional restorations made with two brands of bis-acrylic resin (LuxaCrown^®^ and Protemp™ 4) and to compare the fracture resistance of these two materials. Twenty crowns (ten manufactured using each brand) served as a control, and another forty crowns (twenty of each brand) were aged using artificial saliva with pHs of 4 or 7, for 7 days at 37 °C, in an attempt to simulate the behavior of these materials inside the oral cavity. Subsequently, all crowns were subjected to the application of a force at a constant speed, in a universal testing machine, until fracture was achieved. The LuxaCrown^®^ brand showed greater resistance to fracture than the Protemp™ 4 brand. Salivary pH did not influence the fracture resistance of provisional LuxaCrown^®^ crowns but did influence the fracture resistance of provisional Protemp™ 4 crowns.

## 1. Introduction

Osseointegrated dental implants have revolutionized oral rehabilitation treatments with the aim of restoring edentulous spaces and contributing to improved aesthetics and masticatory function. Rehabilitation with dental implants is an important quality-of-life alternative and is indicated for patients with single, partial or total tooth loss [1,2,3]. The success of implant-supported rehabilitation depends on the osseointegration period, which depends on several factors, including a commonly accepted osseointegration duration between 3 and 6 months [3,4]. This is one of the most important phases in rehabilitation using dental implants, having as a normal consequence the elaboration of a provisional restoration [4]. Temporary restoration plays a fundamental role in oral rehabilitation. It allows patients to rehabilitate during the osseointegration period, restoring function, phonetics and aesthetics, and adapting the patient to the final restoration format [3,5].

Implant-supported provisional restorations (ISPRs) lead to a superior quality of life by contributing to aesthetic improvement, meeting patients’ functional and psychological demands, and facilitating a smooth transition from natural dentition to implant-supported restorations [1,3]. The physiological advantage of a fixed restoration during implant integration is the avoidance of transmucosal loading of the implants and thus avoiding a procedure with a second surgical phase [1].

ISPRs require a temporary abutment, usually made of polyether ether ketone (PEEK) or titanium, to adapt the provisional restoration [4]. ISPRs can be elaborated using direct or indirect methods; elaboration is essential to improve the aesthetics, contour and architectural modeling of the peri-implant tissue. Several authors have described ISPRs prepared using indirect methods, and their advantages and resistance characteristics [2,6,7,8]. However, direct methods can be used to perform an ISPR. The realization of an ISPR using a direct method is an effective, economical treatment with a simple procedure that requires a temporary abutment screwed to the implant and the adaptation of a temporary crown. The provisional restoration can be manufactured using a silicone matrix or a vacuum on a preoperative cast or on an ideally contoured waxing of the replacement tooth [4]. A properly adapted ISPR implies better esthetics, contouring and architectural modeling of the upper peri-implant tissue [9].

Temporary restorations must have an excellent adaptation, as well as a good relationship with the periodontal tissues, and occlusal balance. In this way, effective wear resistance provides an effective balance in these functions. When temporary restorations do not ensure wear resistance, they can lead to changes in the vertical dimension [10].

The resistance of the materials used in provisional restorations is essential to guarantee masticatory efficiency, ensuring that there is an occlusal balance. There are several wear mechanisms that occur in the intraoral tribological process during mastication. These mechanisms are abrasion, adhesion, fatigue and chemical dissolution during wear. The surface finish of temporary restorations is also a fundamental factor that limits material wear. In this way, provisional restorations must guarantee resistance to fracture and wear, promoting more resistant and durable restorations that protect the occlusal and masticatory function [11].

Another factor to take into account in ISPRs is the effect of roughness when selecting the material. Their surfaces must have good finishing and polishing characteristics and thus reduce biofilm and material accumulation. The provisional restorations made with bis-acrylic resins have characteristics that support these assumptions [12]. In 2022, Mondelli et al. evaluated the best polishing protocol for different bis-acrylic resins in comparison with acrylic and composite resins to determine the surface roughness modification and abrasive wear after simulated brushing [12]. The authors, in the study, refer that in bis-acrylic resins, only the removal of the superficial layer with a gauze soaked in alcohol is recommended in addition to the inter-proximal and occlusal adjustments that may be necessary. They also refer that the bis-acrylic resin, namely Protemp^TM^ 4, showed significantly lower roughness values after polishing than the other materials [12].

There are several possible ways to perform an ISPR using a direct method; bis-acrylic resins are commonly used materials. LuxaCrown^®^ (DMG^®^ Chemisch-Pharmazeutische, Hamburg, Germany) is a self-curing composite based on bis-acrylic resin intended for the production of crowns and bridges, consisting of a polymeric matrix, which incorporates 70% silicate glass filling substances and Telio CAD, a polymethylmethacrylate (PMMA). According to DMG^®^ Chemisch-Pharmazeutische, Hamburg, Germany, this material is indicated for temporary or permanent restorations, restoration of temporary crowns and bridges on permanent teeth, implant cases, aesthetic restoration cases, pediatric cases, healthy elderly patients and palliative patients. Protemp™ 4 (3MTM, St. Paul, MN, USA), has been used as a reference in several research studies of bis-acrylic resins used for temporary restorations, such as crowns, bridges, inlays, onlays and veneers [13,14,15]. This material is a self-polymerizable nanoparticulate composite used to manufacture provisional restorations (multiple and single elements); it is composed of dimethacrylates, initiators, plasticizers, pigments and nanometric filler particles (amorphous and colloidal silica).

A dental in vitro investigation should be designed in an environment as similar as possible to the oral cavity environment; creating an experimental analogy to real-world environments contributes to more reliable clinical tests. In this study, saliva function, force and velocity were included to reproduce the oral cavity environment [16].

Human saliva varies in response to several factors throughout the day, such as the ingestion of certain foods that cause bacteria to ferment carbohydrates and a consequent decrease in oral pH. Several studies report that gastroesophageal reflux disease (GERD), one of the most prevalent digestive diseases, can also lead to decreased oral pH [17,18]. One consequence of a decrease in oral pH is the constant contact of crowns’ constituent materials with this environment. Some studies claim that saliva and diet may be responsible for the degradation of various restorative materials [13,19]. Dietary solvents, especially those with acidic components, can penetrate the organic polymeric network of resins causing the filler and matrix phases to swell and separate, followed by softening of the polymers, loss of substance in the oral environment and chemical dissolution. In addition, the deleterious effects of weak intraoral acids (citric and lactic acid) on inorganic fillers can also contribute to a decrease in materials’ hardness [13,19]. Thus, fracture resistance is very relevant and should be considered when choosing material for temporary restorations [9].

In an attempt to simulate the behavior of restorative materials inside the oral cavity, and taking into account the influence of water absorption in bis-acrylic resins, this study used artificial saliva with pHs of 4 and 7 for 7 days in an incubator at 37 °C. However, it is impossible to obtain artificial saliva that accurately reproduces the characteristics of human saliva, which is very inconsistent and unstable [20].

This study aimed to evaluate the influence of oral pH on the fracture resistance of implant-supported provisional restorations made with two brands of bis-acrylic resin (LuxaCrown^®^ and Protemp™ 4) and to compare the fracture resistance of these two materials.

## 2. Materials and Methods

### 2.1. Materials

All materials and chemicals were used in accordance with manufacturers’ standards. This study used two brands of bis-acrylic resins, LuxaCrown^®^ (DMG^®^ Chemisch-Pharmazeutische, Hamburg, Germany) and Protemp™ 4 (3MTM, St. Paul, MN, USA).

### 2.2. Methods

A standard laboratory protocol was established and applied to test all selected samples at the Oral Pathology and Rehabilitation Research Unit, University Institute of Health Sciences (IUCS), CESPU, Gandra, Portugal.

#### 2.2.1. Preparation of the Sample

This study tested 60 temporary bis-acrylic resin crowns, 30 crowns manufactured using LuxaCrown^®^ and 30 crowns manufactured using Protemp™ 4. Sample size calculations were performed with G*Power software (latest ver. 3.1.9.7; Heinrich-Heine-Universität Düsseldorf, Düsseldorf, Germany) and based on a minimum effect size of 0.50, α = 0.05 and 1-β = 0.80. Temporary crowns were prepared via silicone pre-impression. A silicone key was made for a single permanent molar to eliminate possible variables. The 60 crowns were divided into six groups: Group 1 (10 LuxaCrown^®^ crowns in a dry environment, control), Group 2 (10 LuxaCrown^®^ crowns immersed in artificial saliva at pH 4), Group 3 (10 LuxaCrown^®^ crowns immersed in artificial saliva at pH 7), Group 4 (10 Protemp™ 4 crowns in a dry environment, control), Group 5 (10 Protemp™ 4 crowns immersed in artificial saliva at pH 4), and Group 6 (10 Protemp™ 4 crowns immersed in artificial saliva at pH 7).

#### 2.2.2. Elaboration of Temporary Crowns

**Figure 1:**
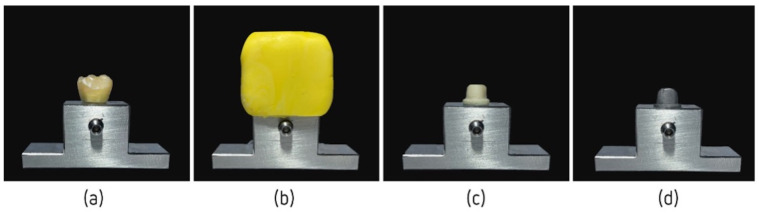
Elaboration of the components: (**a**) first permanent molar adaptation on the transfer table; (**b**) pre-impression of the first permanent molar; (**c**) dental preparation of the tooth previously selected; and (**d**) metallic component corresponding to the provisional abutment.

A titanium transfer table was adapted to the testing machine’s (Instron^®^, Electropuls E10000 Linear-Torsion (Norwood, MA, USA)) support table for the test trials. To ensure that all crowns were the same, a first permanent molar was adapted on the transfer table (Figure 1a) and a pre-impression was performed using silicone (Figure 1b) so that there was no variability in the size of the crowns during execution. Dental preparation was carried out on the previously selected tooth following conventional norms (Figure 1c) to create the support structure for the cementation of the provisional crowns.

Next, digitalization was performed according to the manufacturer’s standards. Scanning was performed using a Planmeca Emerald™ Intraoral Scanner (Planmeca^®^ Oy, Helsinki, Finland). The scan was stored using Planmeca Romexis^®^ software (Planmeca^®^ Oy, Helsinki, Finland) and converted into a standard tessellation language (STS) file. The metallic component corresponding to the provisional abutment was digitally designed using a CAD/CAM system and manufactured using cobalt–chrome by Zirkonzahn^®^ (Gais, South Tyrol, Italy). This component was screwed onto the transfer table, becoming an integral part of it (Figure 1d).

**Figure 2:**
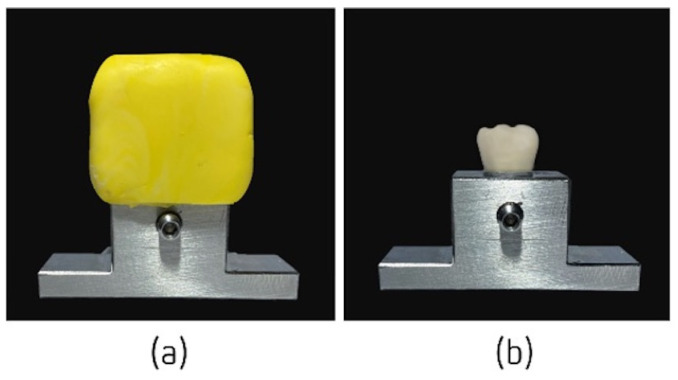
Confection of crowns: (**a**) filled impression on the abutment, and (**b**) crown definitively cemented onto the abutment.

The LuxaCrown^®^ and Protemp™ 4 crowns were prepared according to the manufacturers’ recommendations, on the previously described metallic structure, using the silicone impression made previously. The area corresponding to the abutment was vaselined, preventing the material from adhering, and the silicone impression was filled with bis-acrylic resin. After the materials began to be mixed, the filled impression was placed on the abutment within a maximum period of 40 s, which exerted 1 kg of pressure (Figure 2a). Excess material was removed with a spatula and the provisional crowns were finished and polished. This methodology was repeated 60 times to obtain the necessary samples. The crowns were definitively cemented onto the abutment (Figure 2b) with a composite luting system with dual curing; PermaCem^®^ 2.0 (DMG^®^ Chemisch-Pharmazeutische, Hamburg, Germany) was used for the adhesive fixation of crowns.

**Figure 3:**
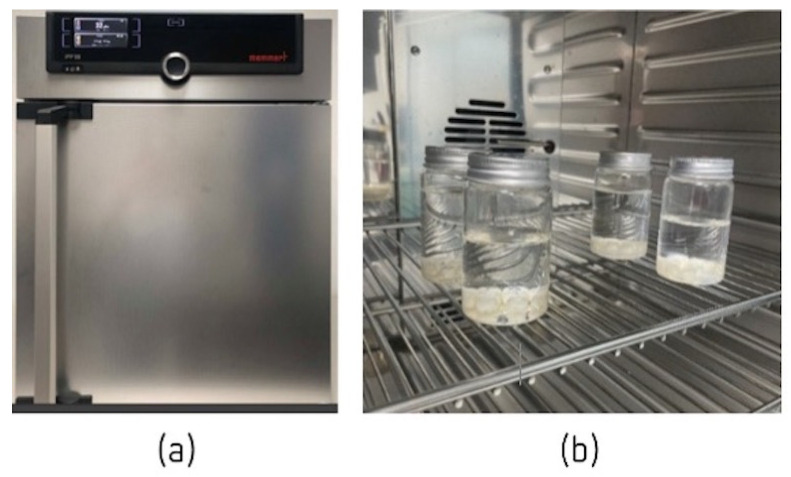
(**a**) IPP110 plus incubator, and (**b**) crowns immersed in artificial saliva with pH values of 4 and 7.

Before starting the mechanical fatigue test, 40 temporary crowns were immersed in artificial saliva with one of two pH values (4 or 7) for 7 days in an IPP110 plus incubator (Memmert^®^, Schwabach, Germany) (Figure 3) at 37 °C to simulate the oral cavity environment [19]. The artificial saliva used was based on the Fusayama Meyer formula (0.4 g/L NaCl, 0.4 g/L KCl, 0.795 g/L CaCl_2_·2H_2_O, 0.005 g/L Na_2_S·9H_2_O, 0.69 g/L NaH_2_PO_4_·2HSO and 1 g/L urea) at 37 ± 2 °C. HCl was incorporated into the base formula to obtain pH values of 4 and 7 [15]. After this exposure, the mechanical strength of the bis-acrylic resins was tested using an Instron^®^ (Electropuls E10000 Linear-Torsion; Norwood, MA, USA) testing machine.

### 2.3. Compression Tests to Measure the Fracture Resistance of Different Temporary Crowns

**Figure 4:**
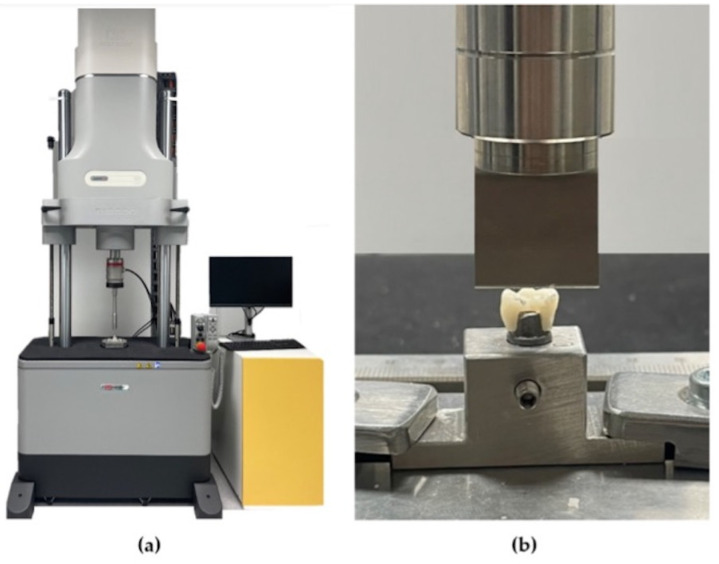
(**a**) Instron^®^ universal testing machine, and (**b**) simulated compressive force movement.

The 40 crowns immersed in artificial saliva and the 20 crowns in the control group were subjected to a simple load test at a constant speed of 1 mm/min in the Instron^®^—Electropuls E10000 LT universal testing machine (Figure 4a). The titanium transfer table, including the structure made for the study and the provisional crown, was fixed to the Instron^®^ fixation support table (Figure 4a). This facilitated its connection to the Instron^®^ Electropuls E10000 LT testing machine, a dynamic fatigue testing machine with a 10 KN linear dynamic capacity, a 7 KN linear static capacity, a 60 mm linear stroke and a 100 Nm torque capacity, which allow static and dynamic axial and torsion tests, in accordance with the ISO 7500-1 standard. It has an accredited calibration force up to 5 meganewtons according to ISO 7500-1 and ASTM E4.

Samples placed on the transfer table were attached to the bottom fixed compartment of the Instron^®^ testing machine. Fracture testing was performed via compressive mode loading applied to the occlusal surface of the temporary crowns using a rectangular metal rod (3.6 mm diameter and 30 mm length) coupled to the load cell of the testing machine at a speed of 1 mm/min (Figure 4b). The rectangular metal rod obtained a homogeneous distribution of stresses on the provisional crowns.

Fracture was detected by an audible crack and confirmed by a sharp drop in the load–deflection curve; test results were recorded using Bluehill^®^ Lite test software version 2.0 (Instron^®^, Norwood, MA, USA), which facilitated the definition and execution of tests and data acquisition. Next, all values and data were transferred to Microsoft Office Excel^®^, version 16.0 (Redmond, WA, USA), which was used to perform statistical data analysis. The loads required for fracture were recorded in Newtons (N).

### 2.4. Statistical Analysis 

Data were analyzed using Environment R 4.1.3 (an open-source statistical R Core Team 2022 program available on the internet for download) [21]. Means (M) and standard deviations (SD) were calculated in the descriptive analysis of the fracture strength of LuxaCrown^®^ and Protemp^TM^ 4 crowns, which considered the dry, pH 4 and pH 7 environments. The Shapiro–Wilk test was used to assess the normality of resistance distributions, considering the 10 observations included for each group. Proof values > 0.05 were obtained for all distributions, making them admissible for parametric statistics. Considering the specific nature of the tests used (one-way ANOVA and factorial ANOVA), the homogeneity of variances was also evaluated using the Levene test, with proof values > 0.05 for all distributions; the null hypothesis of homogeneity of variances was not rejected. Results of a one-way ANOVA were used to compare environments by crown type. Results of a two-way ANOVA were used to evaluate interactions between environments and the factor regarding fracture resistance. Tukey multiple comparison tests were used to compare the environments for each crown type, and LuxaCrown^®^ and Protemp^TM^ 4 crowns. The statistical significance considered for rejection of the null hypothesis was 5%. The effect size was evaluated with eta2 (η^2^) considering the cutoff points 0.01 (small effect), 0.06 (medium effect) and 0.14 (large effect).

## 3. Results

**Figure 5:**
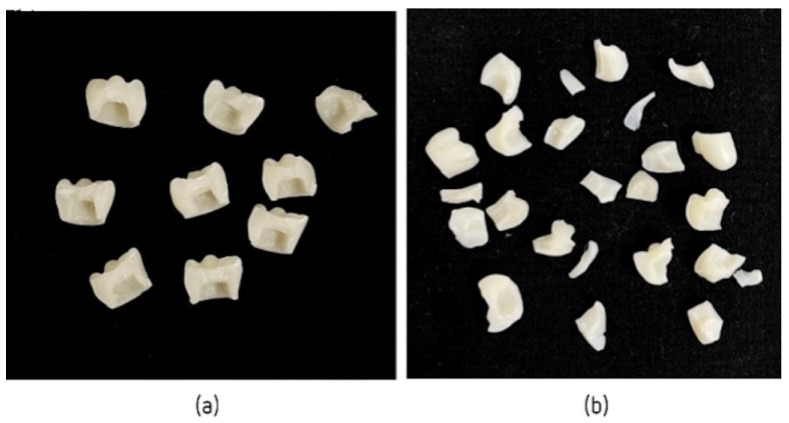
(**a**) LuxaCrown^®^ crowns after mechanical compressive strength test, and (**b**) Protemp^TM^ 4 crowns after mechanical compressive strength test.

All crowns subjected to artificial aging showed no signs of failure or damage. Crowns subjected to the mechanical compression test showed different fractures. After the tests reached maximum strength, LuxaCrown^®^ crowns showed total/partial crown fracture, most often resulting in two symmetrical fragments (Figure 5a). Protemp^TM^ 4 crowns showed a splinter fracture, resulting in several thin, sharp fragments (Figure 5b).

**Figure 6:**
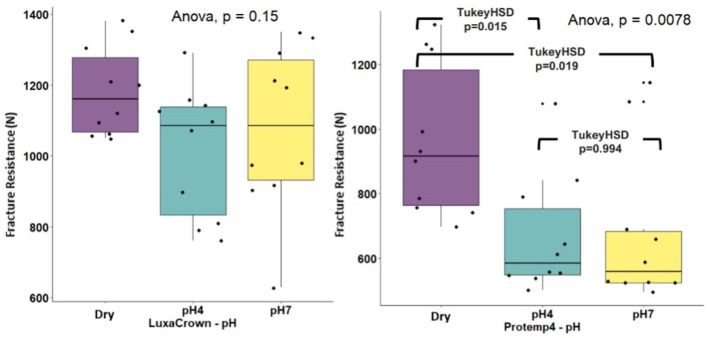
Box and line diagrams for pH comparison by crown type.

**Figure 7:**
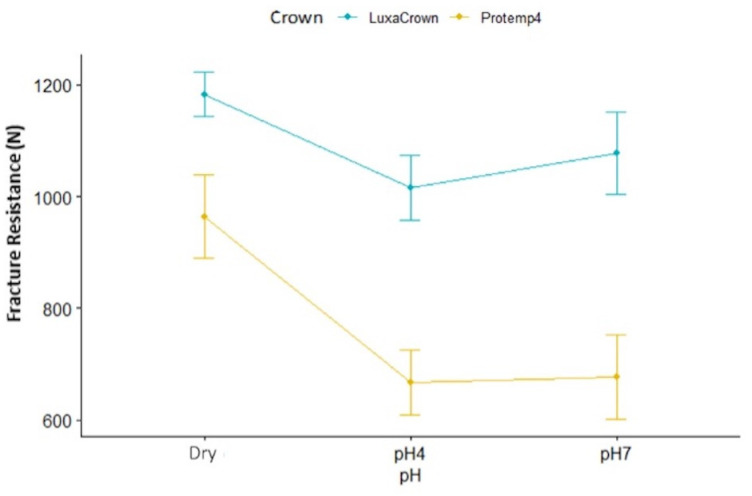
Interaction of pH with crown type regarding fracture strength (N).

Table 1 presents descriptive statistics in M format (DP) for LuxaCrown^®^ and Protemp^TM^ 4 crowns for fracture strengths (expressed in Newtons) by environment (dry, pH 4 and pH 7). Subtotals by environment and crown type (LuxaCrown^®^ and Protemp^TM^ 4) are also presented. The one-way ANOVA test was used to compare strength in three environments stratified by crown type. Statistically significant differences were observed in comparisons of Protemp^TM^ 4 crowns by environment: F(2.27) = 2.048 (*p* = 0.0078) with a high effect size, η^2^ = 0.302, detected differences between the dry medium and pH 4 (*p* = 0.015) and pH 7 (*p* = 0.019) (Figure 6). The average dry strength of Protemp^TM^ 4 crowns was 963,54 (DP = 235,37), which was high compared with the mean strengths obtained at pH 4 (M = 666.66, DP = 182.98) and pH 7 (M = 679.59, DP = 239.44). No statistically significant differences were observed for LuxaCrown^®^ crowns in comparisons of means: F(2.27) = 5.833 (*p* = 0.150), and η^2^ = 0.132. The interaction between crown type and environment was not statistically significant: F(2.54) = 1.051 (*p* = 0.037), and η^2^ = 0.357. In addition, comparison of the subtotals of LuxaCrown^®^ (M = 1091.33, SD = 193.17) and Protemp^TM^ 4 (M = 768.93, SD = 254.92) crowns was statistically significant: F(1.54) = 37,328 (*p* < 0.001), with a high effect size, η^2^ = 0.409, and higher strength in LuxaCrown^®^ crowns, as seen in Figure 7.

**Figure 8:**
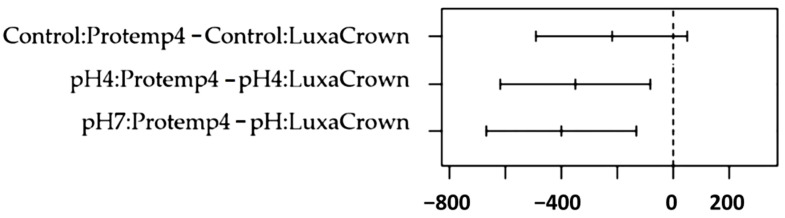
Tukey multiple comparison tests for paired pH comparisons between crown types.

Figure 8 shows Tukey tests comparisons, highlighting comparison results between LuxaCrown^®^ and Protemp^TM^ 4 crowns in dry media, pH 4 and pH 7. Statistical significance was confirmed whenever the confidence interval, observed in Figure 3, did not intersect the difference of 0. Thus, considering the comparisons listed above, statistically significant results were detected between the LuxaCrown^®^ and Protemp^TM^ 4 crowns at pH 4 (*p* = 0.005) and at pH7 (*p* < 0.001); LuxaCrown^®^ crowns had a higher average strength at both pH levels. 

## 4. Discussion

For implant-supported rehabilitations, namely, rehabilitations with implant-supported provisional restorations (ISPRs), the materials’ strength is important, as it implies the integrity of the rehabilitation. In this study, we tested the fracture resistance of two bis-acrylic resins, LuxaCrown^®^ and Protemp™ 4. To accomplish this, 60 provisional crowns were manufactured, 30 using LuxaCrown^®^ and 30 using Protemp™ 4. They were divided into groups of 10, according to aging, in artificial saliva, at pH 4 and pH 7 conditions. This protocol is in line with other studies on resistance to fracture by other authors [22,23]. In 2015, Karaokutan et al. carried out a study that investigated the influence of different fabrication methods and materials on the fracture resistance of different provisional crowns. The authors studied the resistance of six different materials. For each material, ten replicates were reproduced (*n* = 10) by experimental group. The authors also subjected the experimental groups to thermocycling for one week [23].

The study of dental materials implies observing their characteristics and determining whether they benefit patients. Two of the most important aspects in assessing the viability of temporary materials for implant rehabilitation are their durability and fracture resistance. In this study, all crowns were placed in a thermostatic bath at 37 °C for 7 days, a procedure used in several similar studies [16,24]. 

Bite force (BF) is relevant in masticatory function and mastication. Several studies describe maximum BF in normal and parafunctional masticatory function as varying between 244 and 859 N [25,26,27]. Given this, and analyzing our results, we observed that fracturing all LuxaCrown^®^ crowns tested required a force greater than the average reference value. In contrast, only Protemp^TM^ 4 crowns in the control group had fracture values higher than the average maximum masticatory forces reported in the literature. Given this, our results indicate that in terms of prosthetic rehabilitation resistance, LuxaCrown^®^ ISPRs’ fracture resistance was superior to that of Protemp^TM^ 4 ISPRs. In comparison with the mean bite force reported by several authors [25,26,27], the two materials tested in this study, LuxaCrown^®^ and Protemp^TM^ 4, demonstrated average fracture toughness capable of guaranteeing ISPRs.

One of the objectives of this study was to evaluate whether oral pH influenced the fracture resistance of implant-supported provisional restorations made with two brands of bis-acrylic resin. The resistance to fracture of the two bis-acrylic resins, LuxaCrown^®^ and Protemp™ 4, obtained different results, especially when subjected to artificial saliva with pHs of 4 and 7. Fracture strength was superior in LuxaCrown^®^ provisional crowns compared with Protemp™ 4 provisional crowns. It was verified that LuxaCrown^®^ crowns (groups G1, G2 and G3) presented identical behavior in a dry environment (G1) and in artificial saliva with pHs of 4 and 7 (G2 and G3, respectively), demonstrating constant behavior independent of the oral environment. A slight decrease in fracture resistance was observed when the crowns were placed in artificial saliva, with a lower value when at a pH of 4 (G2). However, results indicated that differences at this level were not statistically significant. These results are similar to those of studies that compared the flexural strength of temporary materials based on bis-acrylic resin after conditioning in artificial saliva [15,28]. 

In this study, provisional crowns in LuxaCrown^®^ obtained an average resistance to fracture in a dry environment of 1182 N, which was higher when these crowns were submitted to artificial saliva. When subjected to artificial saliva at a pH of 4, the fracture resistance was 1015 N and when these crowns were subjected to artificial saliva at a pH of 7, the fracture resistance was 1077 N.

Protemp^TM^ 4 crowns (groups G4, G5 and G6) behaved differently after placement in artificial saliva; fracture resistance decreased in crowns aged in artificial saliva at pH 4 (G5) and pH 7 (G6). This decrease in fracture resistance after conditioning in artificial saliva and under acidic pH conditions is similar to the results of other studies [14,19,29,30]. Provisional crowns in Protemp^TM^ 4 obtained an average resistance to fracture in a dry environment of 963.54 N. When subjected to artificial saliva at pH 4, the mean resistance to fracture was 66.66 N, and when subjected to artificial saliva at pH 7, the mean resistance to fracture was 768.93 N. According to Singh et al. (2016), excessive water absorption can promote breakage, causing detachment of the filling matrix [30]. Absorbed molecules, such as water or saliva, separate the polymer chains and facilitate sliding between the chains. After polymerization, a certain amount of unpolymerized monomer can affect mechanical properties and biocompatibility, thus reducing dimensional stability and wear resistance, softening the resinous material [30]. Silane hydrolysis and crack formation can also decrease mechanical properties [13,19]. Koumjian et al. (1990) tested the fracture toughness of seven temporary materials in both dry and wet storage and concluded that fracture toughness values decrease after storage in artificial saliva for seven days because of the plasticizing effects of water [29]. As in our study, the main component of these solvents was water, which may be the cause of this deleterious effect; this decrease in fracture resistance can be explained by the adverse effects of low pH on the inorganic fillers of bis-acrylic resins.

Thus, according to the results obtained in the study, the crowns in LuxaCrown^®^ and Protemp^TM^ 4, when subjected to artificial saliva with pHs of 4 and 7 obtained statistically significant results, in which the provisional crowns in LuxaCrown^®^ obtained a higher average resistance. 

In general, provisional crowns manufactured using LuxaCrown^®^ bis-acrylic resin were more resistant and required a higher load force to fracture and completely fragment; they presented a more homogeneous and linear fracture. Provisional crowns manufactured using Protemp^TM^ 4 bis-acrylic resin fractured more heterogeneously, requiring a lower load force for fracture to occur. Provisional crowns manufactured using Protemp^TM^ 4 bis-acrylic resin fractured more heterogeneously, requiring a lower load force for fracture to occur. Although, all fractures were observed on the surfaces of force applied to crowns made of bis-acrylic resins, LuxaCrown^®^ and Protemp™ 4, and all failures verified were catastrophic, which goes against the study carried out by Karaokutan et al. [23].

This work’s main clinical objective was to determine the basic characteristics of bis-acrylic resins used in oral rehabilitation (obtained by a direct method) to find the best material for a given rehabilitation and patient’s clinical condition. With limitations, this study’s aim was to simulate what could occur in the oral cavity; there are several reasons for a decrease in oral pH, such as consuming sugars, which results in the production of more acid [17]. Although this study simulated several clinical parameters, it is important to recognize that any in vitro study design that intends to reproduce the oral environment’s complex biomechanics, including mastication, has certain limitations; the results must be carefully interpreted.

## 5. Conclusions

Based on the results obtained and according to the methodology described in this study, we formulated the following conclusions:

There were differences in fracture resistance between the two brands of temporary crowns studied. The LuxaCrown^®^ brand showed greater resistance to fracture than the Protemp™ 4 brand.

Salivary pH did not influence the fracture resistance of LuxaCrown^®^ provisional crowns but did influence the fracture resistance of Protemp™ 4 provisional crowns. LuxaCrown^®^ provisional crowns, when submitted to artificial saliva with pHs of 4 and 7, had superior resistance to fracture than Protemp™ 4 provisional crowns.

## Figures and Tables

**Table 1:** Comparisons of the mean fracture strength values of LuxaCrown^®^ and Protemp^TM^ 4 crowns by pH.

Crowns	Control	pH 4	pH 7	Subtotal Crowns	ANOVA * pH	ANOVA ** Crowns × pH
LuxaCrown^®^	1182.00 (125.59)	1015.00 (184.58)	1077.00 (233.57)	1091.33 (193.17)	F_(2_._27)_ = 5.833 (*p* = 0.150) η^2^ = 0.132	F_(2_._54)_ = 1.051 (*p* = 0.357) η^2^ = 0.037
Protemp^TM^ 4	963.54 (235.37)	666.66 (182.98)	679.59 (239.44)	768.93 (254.92)	F_(2_._27)_ = 2.048 (*p* = 0.0078) η^2^ = 0.302	
Subtotal pH	1072.77 (215.11)	840.83 (252.85)	876.80 (308.53)	930.13 (276.96)		

Results presented in M (DP) format of fracture strengths expressed in Newtons; * ANOVA 1 factor; ** Factor ANOVA.

## Data Availability

Data that support this study’s findings are available from the corresponding author upon request.
